# Identification of distinct genotypes in circulating RSV A strains based on variants on the virus replication-associated genes

**DOI:** 10.1101/2024.04.22.590570

**Published:** 2024-04-23

**Authors:** Abdulafiz O. Musa, Sydney R. Faber, Kaitlyn Forrest, Kenneth P. Smith, Shaon Sengupta, Carolina B. López

**Affiliations:** a Department of Molecular Microbiology, Washington University School of Medicine, Saint Louis, Missouri, USA.; b Center for Womeńs Infectious Diseases Research, Washington University School of Medicine, Saint Louis, Missouri, USA.; c Department of Pediatrics, Perelman School of Medicine, University of Pennsylvania, Philadelphia, Pennsylvania, USA.; d Division of Neonatology, Children’s Hospital of Philadelphia, Philadelphia, Pennsylvania, USA.; e Department of Pathology and Laboratory Medicine, Perelman School of Medicine, University of Pennsylvania, Philadelphia, Pennsylvania, USA.; f Infectious Disease Diagnostics Laboratory, Children’s Hospital of Philadelphia, Philadelphia, Pennsylvania, USA.

**Keywords:** respiratory syncytial virus, genotypes, polymerase L, replication-associated genes

## Abstract

Respiratory syncytial virus is a common cause of respiratory infection that often leads to hospitalization of infected younger children and older adults. RSV is classified into two strains, A and B, each with several subgroups or genotypes. One issue with the definition of these subgroups is the lack of a unified method of identification or genotyping. We propose that genotyping strategies based on the genes coding for replication-associated proteins could provide critical information on the replication capacity of the distinct subgroup, while clearly distinguishing genotypes. Here, we analyzed the virus replication-associated genes N, P, M2, and L from *de novo* assembled RSV A sequences obtained from 31 newly sequenced samples from hospitalized patients in Philadelphia and 78 additional publicly available sequences from different geographic locations within the US. In-depth analysis and annotation of the protein variants in L and the other replication-associated proteins N, P, M2-1, and M2-2 identified the polymerase protein L as a robust target for genotyping RSV subgroups. Importantly, our analysis revealed non-synonymous variations in L that were consistently accompanied by conserved changes in its co-factor P or the M2-2 protein, suggesting associations and interactions between specific domains of these proteins. These results highlight L as an alternative to other RSV genotyping targets and demonstrate the value of in-depth analyses and annotations of RSV sequences as it can serve as a foundation for subsequent *in vitro* and clinical studies on the efficiency of the polymerase and fitness of different virus isolates.

## INTRODUCTION

Respiratory Syncytial Virus is a negative-sense single-stranded RNA virus that belongs to the family *Pneumoviridae* and order Mononegavirales. RSV circulates seasonally around the world, and it is estimated to infect every child once before the age of three, with the possibility of reinfections throughout life(1, 2). RSV leads to a wide variety of clinical outcomes that range from a mild cold to bronchiolitis, pneumonia, and death(2). RSV is a leading cause of hospitalizations in children and a major cause of morbidity in adults(3, 4). Each year, in the United States (US) alone, it is estimated to be the cause of 58,000–80,000 hospitalizations for children under the age of 5(5, 6). Recent advances in anti-RSV antivirals and vaccines for selected populations are encouraging(7–12). However, given the circulating nature of RSV among the human population, the abundance of infections, and the wide variety of clinical manifestations, there is a need for a better understanding of the genomic determinants of RSV pathogenesis. A key step in this direction is to improve the identification of RSV genotypes that are associated with different degrees of virus replication and pathogenicity.

The RSV genome is composed of 10 genes NS1, NS2, N, P, M, SH, G, F, M2, and L that encode 11 proteins. The M2 gene contains two overlapping open reading frames that code for two proteins, M2-1 and M2-2(13). The initial stage of RSV infection is determined by the virus surface glycoproteins G and F that mediate the binding of the virus to its receptor and fusion with the host cell membrane, respectively(14). G protein undergoes high selective pressure, causing frequent variations between genomes(15). The F protein, also found on the surface of the virus, is fairly conserved among different RSV strains(14, 15). Once the virus enters a cell, virus transcription, and genome replication are mediated by the polymerase L and its co-factors, including P, M2-1, and the nucleoprotein N(13, 16). M2-2 has been shown to play a role in the switch between transcription and replication, suggesting a role with the ribonucleocapsid complex(17–19).

Since its first discovery in 1956(20, 21), RSV has been classified into two main serotypes, RSV-A and RSV-B(22, 23), both of which can be further divided into multiple genotypes. Historically, due to its relatively smaller size and high variability, the evolutionary events in the G gene have been used to genotype RSV. In most cases, RSV genotypes can be distinctly defined based on the 2nd hypervariable region (HVR2) or ectodomain of G(24–29). Because G variations may impact receptor binding, G-based genotype is a good method to identify variants with different entry abilities. Whole genome sequencing has more recently been reported as an alternative method for genotyping, and it has been shown to better represent RSV genotypes in the population(30–33). M2-2 sequencing has also been proposed as an alternative genotyping method, as this is one of the smallest viral proteins with a low level of conservation(34). Notably, no current routine genotyping method focuses on the viral polymerase and its associated genes, despite the critical role of these proteins in viral replication and infection.

The lack of consensus on how to determine RSV genotypes makes it challenging to track circulating strains among the various reported sequences and even more difficult to predict associations with the replicative capacity of the different RSV variants. To assess whether variations in the replication-associated proteins can identify RSV genotypes, we took an in-depth look at the variants present in *de novo* assembled whole-length RSV genomes circulating in the US between 2012–2023. We included 31 newly sequenced samples obtained from a cohort of hospitalized children in Philadelphia, as well as 78 publicly available data sets of RSV A sequences obtained from samples around the country. Comparing RSV A sequences from samples in different US regions between the years 2012–2023, we found predictable RSV A genotypes distinguished solely by variants on the L gene. Remarkably, non-synonymous variations in L were consistently accompanied by conserved changes in the L co-factors P or M2-2, suggesting the co-variation of replication-association proteins in circulating genotypes. Furthermore, these non-synonymous variations accurately predicted RSV genotypes based on the whole genome sequence. This report demonstrates the importance of in-depth analysis of full-length RSV sequences to uncover potentially important functional changes in viral proteins, and it identifies clusters of associated variations in polymerase-related genes that can be used to genotype RSV with high accuracy.

## RESULTS

### The G and L genes are the most variable among RSV A variants circulating in the USA during 2012 – 2023.

**Figure 1:**
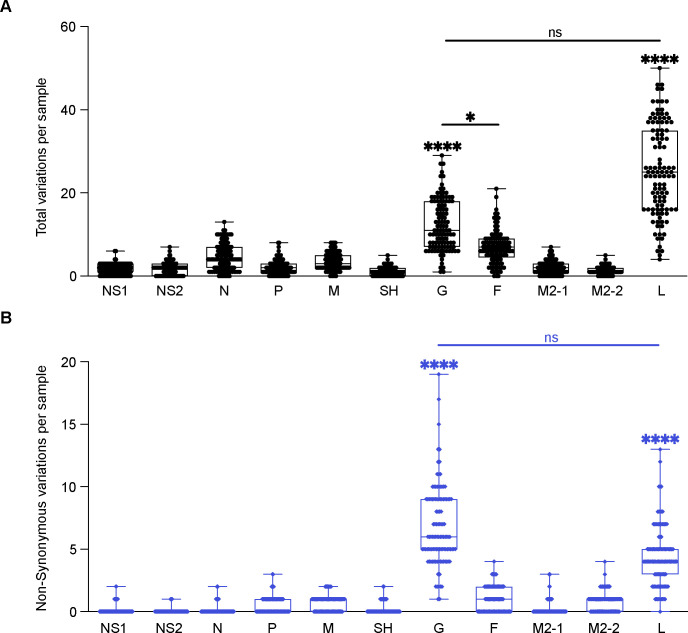
Summary of genetic variations of RSV A 11 CDS regions. Each point represents the number of variants per sample, N=109. **(A)** Total variations per sample for each CDS region. **(B)** Non-synonymous variations per sample for each CDS region. One-way analysis of variance (ANOVA) non-parametric Kruskal-Wallis tests were performed for statistical significance between CDS regions. ns p>0.1234; *p>0.03; ****p<0.0001. Comparisons between all the CDS regions to G or L were **** unless otherwise noted by a line between CDS regions.

First, to identify the most variable genes in recently circulating RSV A variants, we initially sequenced and *de novo* assembled full-length genomes of 31 samples obtained from pediatric patients at the Children’s Hospital of Philadelphia (CHOP). We completely annotated these sequences and submitted them under GenBank: PP525296–PP525326. Using the consensus of the samples as a reference to identify variants between the different samples, we observed the highest number of total variations, and non-synonymous variations, in the coding regions of G and L. The M2-2 coding region showed the highest ratio of non-synonymous to total variation, and NS2 was the most conserved (Table S1). We expanded our dataset with 78 additional RSV A sequences from the National Center of Biotechnology Information (NCBI) database that accounted for different locations in the US as well as an increased range of years the sequences were collected (Table 1 and Table S2). In the combined cohort of 109 RSV A full-length sequences, we again observed the most total and non-synonymous variations in the G and L genes with the M2-2 showing the highest ratio of non-synonymous to total variation (Table 2 and Figure 1).

### Non-synonymous variations in the L gene associate with specific variations in other replication-related genes which can predict genotypic clusters.

We next took a closer look at the non-synonymous variations across the coding regions of the replication-related genes (N, P, M2-1, M2-2, and L) in the 109 sequences and annotated the non-synonymous variations for each sample compared against the consensus sequence (Table S3). We found associations of variations between L and one or more of the other replication genes. We marked out the similar variations in each coding sequence (CDS) region that appeared more than two times and categorized the sequences into groups named R1-R6 based on the observed associations (Table 3, Table S4). Each group contained a varying number of sequences independent of year or location (Table S5).

We separated the sequences in each group based on the variation patterns observed (Table 4). R1 group contained a diverse set of variations. However, most of the R1 sequences contained L: G1725E which associated 44% of the time with M2-2: N46S. Within the sequences containing L: G1725E, three sequences had an additional L variation, N215S, which associated 100% of the time with variants, P: P34L and M2-2: N46S. Sequences with additional L variation, V335I associated 50% of the time with the P variant, I66T. The variant patterns in the R2 and R4 groups were more consistent as at least 75% of the sequences had clear associations of variations in P, M2-2, and L. Interestingly, the R3 group essentially contained L: N143D, T179S, I1653V, K1661N of which 78% did not associate with any variants in P, M2-1, or M2-2. The remaining percentage of the group did however associate with either M2-2: L37P (13%) or a trio of M2-1 variants: N174S, T180A, S182G (9%). Out of the entire cohort analyzed, R3 was the only group to consistently contain variants in M2-1. Although the R5 group had a limited number of sequences, all the sequences in this group were composed of the variants P: N59S, and L:R511K, R1759K.

**Figure 2:**
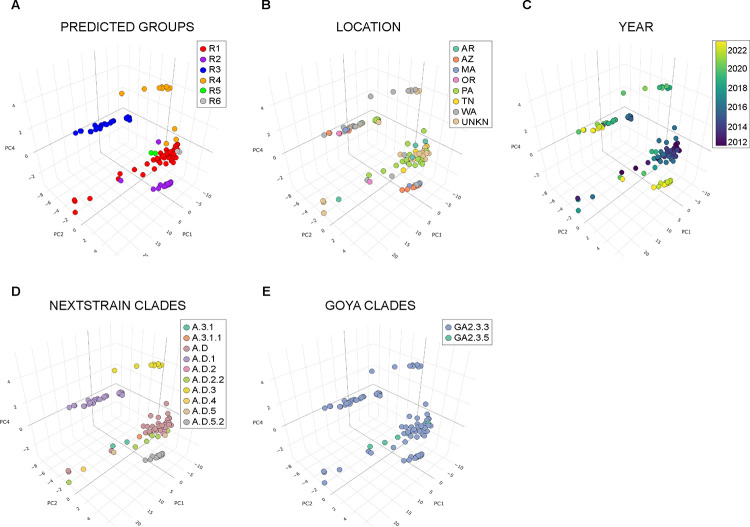
Principal component analysis (PCA) of the 109 RSV A sequences analyzed in this study. Data points were labeled based on (**A-E)** Predicted groups, Location in the US, Year the sample was collected, Nextstrain Clades classification, and Goya Clades classification respectively. The percentage of variance in all the plots are PC1=58.71%, PC2=21.78%, and PC4=2.52%.

Next, we generated a PCA plot with the uncorrected pairwise distances of the 109 full-length sequences and labeled them with our predicted genotypic groups R1-R6 (Figure 2A). We observed that most of the sequences distinctly clustered together based on the pre-determined groups, which supports the predicted associations among related sequences. Additionally, we labeled each sequence by the region or the year the sequences were obtained and found them to be independent of the associations (Figure 2B, C).

We also employed the Nextclade web tool to assign genotypes and depict the phylogeny of the 109 samples based on 2 recently proposed classifications – Nextstrain Clades and Goya Clades(33, 35, 36). The Nextstrain Clades classification resulted in 10 clades and the Goya Clade classification resulted in only two 2 clades, GA2.3.3 and GA2.3.5 (Table S6). We labeled the previously shown PCA plot (Figure 2A) with both classification assignments from Nextclade (Figure 2D, E). Even though there are limitations to the number of clusters that can be visualized on the PCA, we found the Nextstrain Clade classification to be similar to our predicted groups. These results confirm that the non-synonymous variations in the replication-associated genes can be used to identify genotypes among RSV sequences.

### Non-synonymous variations in replication-associated genes concentrate in protein-protein interaction domains.

**Figure 3:**
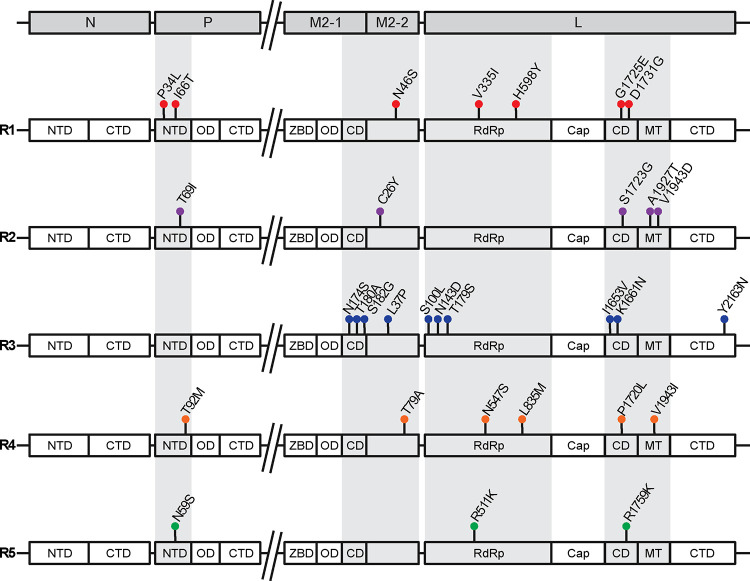
Annotation of non-synonymous variations observed within the domains RSV A replication-associated genes for each predicted group. All variations shown are seen at least 3 times and interact with other variations within a predicted group. Abbreviation key as follows: NTD: N-terminal domain; CTD: C-terminal domain; OD: oligomerization domain; ZBD: Zinc-binding domain; M2-1 CD: core domain; RdRP: RNA-dependent RNA polymerase; Cap: cap addition domain; L CD: connector domain; MT: cap methylation domain.

To determine if specific domains of the RSV replication-associated proteins were susceptible to variation, we annotated the variants observed in the different groups to their respective predicted domain residues (Figure 3)(16). All the variants observed in P were found in the N-terminal domain (NTD). The NTD of P is thought to bind to M2-1, specifically regions of the core domain, as well as interact with RNA-free N monomers(16). The variants of M2-1 were limited to the core domain. The structure of M2-2 has yet to be resolved, therefore no domains are assigned to the protein. However, it has been shown that overexpression of M2-2 rearranges the ribonucleocapsid complex, suggesting potential interactions with L(19). We observed most variants of L in the RNA-dependent RNA-polymerase (RdRp), connector domain (CD), and methyltransferase (MT) domains. It has been shown that the RdRp of L is an interaction site for P(37, 38). The complete structures of the CD and MT domains have not been determined in the attempts to resolve the full L structure; however, it is suggested that these domains may be dynamic and also interact with the P protein(16, 37, 38). Overall, these associations indicate potential residues of interest in the replication-associated proteins that may impact the fitness of the virus during an infection.

## DISCUSSION

Our in-depth analysis of RSV A sequences shows distinct clusters of variations within replication-associated genes as specific variation(s) in L often associate with variations in one or more of the other replication-associated genes. We grouped these associations, depicted them in a PCA plot, and compared their similarity to relatively known methods of RSV genotyping with the Nextclade tool. Our observed associations were not only capable of defining genotypic groups, but also support the advantage of full-length sequence analyses to properly characterize and genotype RSV subgroups. Importantly, our analysis revealed associations of variants among different replication-associated genes. These data can be used to infer the dynamic variation of RSV genotypes and their associated pathogenicity in different seasons, as well as as starting point for detailed investigation of the molecular interactions among proteins from the replication complex.

Most of the reported sequencing analyses from clinical studies generate new sequences based on a known or closely related reference sequence(39–42). An alternative method is *de novo* assembly. The advantage of *de novo* assembly is that it does not require a reference sequence that could potentially introduce bias in the form of variations in the consensus sequence. A merger known as reference-guided *de novo* assembly has also been proposed for larger genome construction (43). In this study, we employed the SPAdes *de novo* assembler with rnaviralSPAdes option which is well adapted for shorter reads and metavirome (44, 45). This gives an accurate sequence per isolate as genomic variations including intergenic, or repetitive regions, and indels are also accounted for.

With *de novo* assembly one must be conscientious when trimming the CDS regions. For RSV, one of the prominent characteristics observed during alignment is the novel duplication in the C-terminal region of the attachment glycoprotein(25). Of the 109 sequences analyzed in this study, only 10 were without the duplication (GenBank: KY967362, MF001039, PP525326, MN306050, MN306054, PP525323, OK659680, PP525325, PP5253324, MN310477). In addition to the duplication event in the G, we also observed the presence of alternate codons while trimming the CDS regions. We observed that these alternate codons often lead to a few extra or lesser amino acidd that may not be properly annotated during database submissions. Observed alternate codons include 7 sequences that have a later stop codon in the G gene (GenBank: OK649680-84, 0R287918, OR287948). This alternate codon is also present in the well-studied reference genome – GenBank: KT992094.1. Two sequences were also observed to have a later stop codon in the L gene in this study, which has not been previously reported (GenBank: PP525304, OR143185). Other sequences without these later stop codons often have a tandem stop codon e.g. “TAATGA”. These tandem stop codons have been studied in the RSV G gene and shown to decrease the expression of fusion glycoprotein (F) and facilitate immune evasion of the virus resulting in the severity of the infection(46, 47). Nine sequences in this study have an alternative start codon in the M2-2 gene (GenBank: PP525314, PP525323-4, OK649682, OK649684, OR143171, OR143187, OR522529, OR601479). M2-2 is generally known to have three start codons and HRSV A2 has this variation too(18, 48). Only the first and second of these start codons have been shown to result in a functional M2-2 protein during replication(18). Alternate start codons in the Influenza virus have been shown not to affect viral fitness, particularly when the translated protein does not result in a missing domain(49, 50).

One unclear question is which protein(s) is/are the major driver of genomic variations: the surface proteins – SH, G, and F that are subjected to host immune activities or the polymerase protein L responsible for ensuring viral growth fitness. The answer could be both. It has been established that the 2nd hypervariable region of G (HVR2) is highly variable and often used in determining RSV genotypes(51). However, in this study, we focused on a replication aspect starting with the polymerase L and observed variations majorly in the RdRP, CD, and MT domains. Consistent associations were also observed in other replication-associated genes such as the NTD of P, the core domain of M2-1, and M2-2. It is known that L, but not P, interacts directly with the RNA template and P interacts with L as a cofactor during replication (52). Established protein prediction also shows that L binds to the CTD of P rather than its NTD(16, 38). However, in the well-studied Vesicular Stomatitis Virus (VSV), it has been shown that the NTD of P interacts with the CTD, RdRp, and CD domains of L(53–55). The CD, MT, and CTD in VSV L have no fixed position and it is this compact conformation with P that transforms it to an initiation competent state(53). With such an interactive polymerase complex there are multiple ways the variations observed may impact the functionality of the virus, such as speed or fidelity. These factors should also be considered when tracking and tracing different RSV genotypes.

With the prevalence of circulating RSV among the human population, it is crucial to understand the genomic determinants of RSV pathogenesis. It has been shown that different RSV strains may impact clinical outcomes(47, 56). Yet, there is a lack of consensus on the exact strains that cause the different severities of disease (57). Improving the annotation of variations specifically involved in virus replication may aid in the identification of RSV genotypes that correspond to pathogenesis. From our results, we support that full-length sequencing is the most reliable data required for genotyping RSV A, closely followed by genotyping the L gene. We also suggest investigating the variation patterns in the CDS of replication-associated genes can be very informative in downstream studies on the efficiency of the polymerase.

## MATERIALS AND METHODS

### Description of cohort and Data collection

Ninety-six RSV clinical samples were obtained from the Children’s Hospital of Philadelphia (CHOP), Philadelphia, Pennsylvania, USA. Samples used in this study were from 3 different coded cohorts. Cohort H (7 samples; 2012 and 2017), Cohort CL (6 samples; 2013 – 2015), and Cohort B (83 samples; 2015 and 2016). All clinical samples were nasal washes collected from hospitalized patients between the age of 0–2 years as reviewed and approved by the CHOP Institutional Review Board (IRB). From the 96 samples, we could *de novo* assemble full-length sequences for the 31 included in this study. Other sample sequences analyzed outside these cohorts were obtained from the National Center for Biotechnology Information (NCBI Virus) and Bacterial and Viral Bioinformatics Resource Center (BV-BRC) databases (78 sequences; 2012 – 2023). Complete sequences without insertions in their CDS were selected at random to increase the number of sequences we have each year (Table S2).

### RNA extraction, library preparation, and NGS sequencing

The processing of samples in Cohort H (7 samples) and CL (6 samples) has been previously described in detail(58, 59). For Cohort B (83 samples), total RNA was extracted from clinical samples using TRIzol LS (Invitrogen) according to the manufacturer’s instructions. Linear Acrylamide (Invitrogen) was added at the precipitation step of RNA extraction to increase yield. RNA quantity and quality were measured using Nanodrop and Bioanalyzer (Agilent Technologies).

Sigma SeqPlex RNA Amplification Kit was used for making the complementary deoxyribonucleic acid (cDNA) library preparation for all samples to be sequenced using the Illumina NovaSeq 6000 to generate 150-bp, paired-end reads. The sequencing generated an average of 50 million reads per sample with an average Phred quality score between 34.6 and 36.1.

### De novo assembly

Illumina adaptors were removed from the resulting Illumina paired-end reads using Cutadapt (v2.5)(60) and the trimmed reads were analyzed using FastQC (v0.11) to ensure their quality. Bowtie2 (v2.4.1)(61) was then used to remove trimmed reads that aligned to the human genome (GRCh38) to generate non-host reads for subsequent analysis. St. Petersburg genome assembler (SPAdes) v3.15.5(44) was used for the de novo assembly of the non-host paired-end reads of each sample. An additional pipeline option in the assembler recommended for RNA viral datasets (--rnaviral) and a thread of 50 were included as parameters while running the assembler with docker on the command line. The contigs and scaffolds generated by the assembler for each sample were aligned against Genbank: KC731482.1 using BLASTN to identify and obtain the RSV A sequences used in this study.

### Variant analysis

MegAlign Pro v17.5.0 (DNASTAR) was used for most of the analysis of the selected sequences of RSV A in this study. The complete nucleotide sequences of all samples were aligned with GenBank: KC731482.1, KT992094.1 using MUSCLE with default options. The coding sequences (CDS) of RSV A (NS1, NS2, N, M, P, G, F, SH, M2-1, M2-2, and L) were obtained by trimming the aligned sequences according to the annotation of the references from their feature tables on GenBank. EMBOSS Transeq tool (EMBL-EBI)(62) was used to translate the trimmed CDS to amino acids. The resulting amino acid sequences for each gene were re-uploaded and re-aligned with MUSCLE, and the reference sequences used in trimming the CDS were excluded from subsequent analysis. Variant calling for each CDS was generated using “Compute Variants” in MegAlign Pro v17.5.0 after which these were compiled as reference amino acid, (X) reference position, (123) variant in the sample (Y) i.e. X123Y. The reference used in this analysis is the consensus sequence generated after the alignment of all the sequences.

### PCA

Principal component analysis graphs were plotted in R Studio v 2023.12.1+402 using plotly library. The input data were the demographics of the sequences and their uncorrected pairwise distance matrix.

### Clades assignment

Assignment of Nextstrain and Goya clades were made in Nextclade v3.2.0 https://clades.nextstrain.org(36).

## Supplementary Material

Supplement 1

Supplement 2

Supplement 3

Supplement 4

## Figures and Tables

**Table 1.** Distribution of the 109 sequences used in this study including the years samples were collected and their locations by states. All 31 PA samples are from the CHOP B Cohort and other sequences were randomly selected from NCBI. UNKN indicates that sequences are of unknown origin within the US.

		US Location								
		AR	AZ	MA	OR	PA	TN	WA	UNKN	Total
**Year**	2012				1	3	2		2	**8**
	2013					7			1	**8**
	2014				2	6			2	**10**
	2015			1		3		4	3	**11**
	2016			3		9		5	3	**20**
	2017		1			1			2	**4**
	2018					1		1	2	**4**
	2019	1	2						7	**10**
	2020	2	2			1			5	**10**
	2021							1	3	**4**
	2022	1	1				1	7		**10**
	2023	1	3					5	1	**10**
	Total	**5**	**9**	**4**	**3**	**31**	**3**	**23**	**31**	**109**

**Table 2.** Computed variations for all 109 samples showing the combined number of substitutions, insertions, and deletions per gene. Total variations were deduced from the nucleotide sequence alignment, and non-synonymous variations were deduced from the amino acid alignment.

Gene	Total number of Variations	Number of Non-Synonymous Variations	Non-synonymous/Total Variations
**NS1**	178	15	0.084
**NS2**	190	6	0.032
**N**	493	15	0.030
**P**	220	56	0.255
**M**	376	73	0.194
**SH**	120	24	0.200
**G**	1348	743	0.551
**F**	749	107	0.143
**M2-1**	219	27	0.123
**M2-2**	145	86	0.593
**L**	2776	486	0.175

**Table 3.** Definition of groups based on repetitive non-synonymous variations often seen in combination across replication-associated genes. Variations included are observed in more than 2 sequences and the groups shown account for all 109 sequences. N is not included here as there is no significant recurrence of non-synonymous variations observed. Within each CDS, a slash “/” indicates that this is another variation seen in the group. For detailed associations, see Table S4.

Group Name	Variations in P	Variations in M2-1	Variations in M2-2	Variations in L
**R1**	I66T/P34L		N46S	G1725E/H598Y/D1731G/V335I/N215S
**R2**	T69I		C26Y	S1723G/A1927T/V1943D/1139T/T179A
**R3**		N174S/T180A/S182G	L37P	N143D/T179S/I1653V/K1661N/S100L/L953M/S1593N/S1789F/H1707Q/A2014/Y2163N
**R4**	T92M		T79A	L835M/A1537S/N547S/P1720L/V1943I
**R5**	N59S			R511K/R1759K
**R6**				No variations

**Table 4.** Detailed variation patterns observed in each group. N is not included here as there is no significant recurrence of non-synonymous variations observed.

Group Name	Variations in P	Variations in M2-1	Variations in M2-2	Variations in L	Number of Sequences
**R1**				G1725E	12
			N46S	G1725E	7
			N46S	H598Y, G1725E, D1731G	4
	P34L		N46S	N215S, G1725E	3
			N46S		3
				H597Y, G1725E	3
			N46S	H598Y, D1731G	2
				V335I, G1725E	2
				H598Y	2
	I66T			V335I, G1725E	2
	I66T			V335I	1
			N46S	H598Y, G1725E	1
				V335I	1
**R2**	T69I		C26Y	S1723G, A1927T, V1943D	9
	T69I		C26Y	I139T, S1723G, A1927T, V1943D	3
	T69I		C26Y	T179A, S1723G, A1927T, V1943D	1
	T69I		C26Y	S1723G, V1943D	1
				S1723G, V1943D	1
				T179A, S1723G, V1943D	1
				T179A	1
**R3**				N143D, T179S, I1653V, K1661N	12
			L37P	N143D, T179S, I1653V, K1661N	4
				N143D, T179S, S1593N, I1653V, K1661N, S1789F, Y2163N	4
		N174S, T180A, S182G		N143D, T179S, I1653V, K1661N	3
				S100L, N143D, T179S, L935M, I1653V, K1661N	3
				N143D, T179S, I1653V, K1661N, A2014T, Y2163N	2
				S100L, N143D, T179S, L935M, I1653V, K1661N, H1707Q	2
				N143D, T179S, I1653V, K1661N, A2014T	1
				S100L, T179S, L935M, I1653V, K1661N, H1707Q	1
**R4**	T92M		T79A	L835M	6
	T92M		T79A	A173S, L835M	3
	T92M			N547S, L835M, P1720L, V1941I	3
				
**R5**	N59S			R511K, R1759K	3
**R6**					2

## Data Availability

All raw sequencing data used for *de novo* assembly were deposited in SRA under accession numbers PRJNA837014, and PRJNA681672. Assembled and annotated sequences were submitted in GenBank and were assigned accession numbers PP525296–PP525326.

## IMPORTANCE

Given the historical heterogeneity of Respiratory Syncytial Virus (RSV) and the disease it causes, there is a need to understand the circulating RSV strains each season. This requires an informative and consensus method of genotype definition. Here, we carried out a variant analysis that shows a pattern of specific variations among the replication-associated genes of RSV A across different seasons. Interestingly, these variation patterns point to previously defined interactions of domains within these genes, suggesting co-variation in the replication-associated genes. Our results also suggest a genotyping strategy that can prove to be particularly important in understanding the genotype-phenotype correlation in the era of RSV vaccination, where selective pressure on the virus to evolve is anticipated. More importantly, the categorization of RSV strains based on these patterns may prove to be of prognostic value.
