# Supplementary material for: Identification of distinct genotypes in circulating RSV A strains based on variants on the virus replication-associated genes
Source: bioRxiv. 2024 Apr 23:2024.04.22.590570. Preprint. [Version 2] doi: 10.1101/2024.04.22.590570 (PMC11071361; doi:10.1101/2024.04.22.590570)
Supplement: Supplement 4 [file NIHPP2024.04.22.590570v2-supplement-4.pdf]

bioRxiv preprint doi: https://doi.org/10.1101/2024.04.22.590570; this version posted April 23, 2024. The copyright holder for this preprint (which was not certified by peer review) is the author/funder, who has granted bioRxiv a license to display the preprint in perpetuity. It is made available under aCC-BY 4.0 International license.

SUPPLEMENTARY TABLES AND LEGENDS:

Table S1: Variations in the 31 RSV A sequences from Philadelphia. Computed variations showing the combined number of substitutions, insertions, and deletions per gene. Total variations were deduced from the nucleotide sequence alignment, and non-synonymous variations were deduced from the amino acid alignment.

	Total Number of Variations	Number of Non-Synonymous Variations	Non-Synonymous/Total Variations
NS1	60	5	8.33
NS2	45	0	0.00
N	120	3	2.50
P	58	10	17.24
M	81	19	23.46
SH	23	2	8.70
G	287	181	63.07
F	195	19	9.74
M2-1	38	6	15.79
M2-2	30	21	70.00
L	709	130	18.34

bioRxiv preprint doi: https://doi.org/10.1101/2024.04.22.590570; this version posted April 23, 2024. The copyright holder for this preprint (which was not certified by peer review) is the author/funder, who has granted bioRxiv a license to display the preprint in perpetuity. It is made available under aCC-BY 4.0 International license.

Table S2: GenBank accession number, length of genome, US states, year of sample collection of all 109 RSV A sequences. UNKN indicates sequences of unknown origin within the US.

Accession Number	Length of genome (bp)	US State	Collection Date
h66_lcl|Query_59835	15248	PA	2012
h7_lcl|Query_91531-rc	15332	PA	2012
h75_lcl|Query_44347-rc	15251	PA	2012
OK649680	15177	US - UNKN	2012
OK649681	15225	US - UNKN	2012
OK649682	15242	US - UNKN	2012
OR466338	15330	US - UNKN	2012
OR466339	15261	US - UNKN	2012
cl79_lcl|Query_82317-rc	15017	PA	2013
KU950506	15202	US - UNKN	2013
KX894805	15173	US - UNKN	2013
KY982516	15218	TN	2013
MN531557	15063	US - UNKN	2013
OR466340	15258	US - UNKN	2013
OR466360	15258	US - UNKN	2013
OR466361	15390	US - UNKN	2013
cl99_lcl|Query_27301-rc	15060	PA	2014
KU839637	15173	TN	2014
KU950464	15232	US - UNKN	2014
KU950523	15226	US - UNKN	2014
KU950537	15231	US - UNKN	2014
KU950686	15231	US - UNKN	2014
LC474556	15228	US - UNKN	2014
LC474557	15231	US - UNKN	2014
LC474558	15231	US - UNKN	2014
OK649683	15242	US - UNKN	2014
b10_lcl|Query_60783	15067	PA	2015
b15_lcl|Query_23936	15118	PA	2015
b2_lcl|Query_82755	15090	PA	2015
b23_lcl|Query_50431-rc	15080	PA	2015
b26_lcl|Query_131352-rc	15066	PA	2015

bioRxiv preprint doi: https://doi.org/10.1101/2024.04.22.590570; this version posted April 23, 2024. The copyright holder for this preprint (which was not certified by peer review) is the author/funder, who has granted bioRxiv a license to display the preprint in perpetuity. It is made available under aCC-BY 4.0 International license.

b5_lcl|Query_50351	15069	PA	2015
cl59_lcl|Query_52150-rc	15108	PA	2015
KY967362	15117	US - UNKN	2015
KY967363	15203	US - UNKN	2015
MF001039	14981	US - UNKN	2015
OK649684	15253	US - UNKN	2015
b30_lcl|Query_44628-rc	15062	PA	2016
b32_lcl|Query_380810	15070	PA	2016
b34_lcl|Query_28812	15111	PA	2016
b36_lcl|Query_65777-rc	15070	PA	2016
b37_lcl|Query_99620	15021	PA	2016
b39_lcl|Query_38058	15063	PA	2016
b42_lcl|Query_76864-rc	15075	PA	2016
b43_lcl|Query_118515	15179	PA	2016
b48_lcl|Query_30126-rc	15112	PA	2016
b60_lcl|Query_68232-rc	15056	PA	2016
b63_lcl|Query_85023	15167	PA	2016
b65_lcl|Query_52294-rc	15077	PA	2016
b66_lcl|Query_251188-rc	15127	PA	2016
b75_lcl|Query_46678	15100	PA	2016
b80_lcl|Query_28470-rc	15107	PA	2016
MN630093	15178	AR	2016
MN630096	15141	AR	2016
MN630098	15102	AR	2016
MN630105	15067	AR	2016
MN630106	15160	AR	2016
h81_lcl|Query_50584-rc	15330	PA	2017
h82_lcl|Query_27979	15252	PA	2017
h84_lcl|Query_51805	15361	PA	2017
h86_lcl|Query_16133	15258	PA	2017
MN306017	15260	US - UNKN	2018
MN306021	15236	US - UNKN	2018
MN310477	15129	US - UNKN	2018
MW033958	15267	TN	2018
MN306029	15191	US - UNKN	2019
MN306050	15156	US - UNKN	2019
MN306054	15116	US - UNKN	2019
ON729318	15233	US - UNKN	2019

bioRxiv preprint doi: https://doi.org/10.1101/2024.04.22.590570; this version posted April 23, 2024. The copyright holder for this preprint (which was not certified by peer review) is the author/funder, who has granted bioRxiv a license to display the preprint in perpetuity. It is made available under aCC-BY 4.0 International license.

ON729319	15171	US - UNKN	2019
OR287841	15137	WA	2019
OR287842	15134	WA	2019
OR287846	15197	WA	2019
OR287849	15196	WA	2019
OR287859	15197	WA	2019
OQ331220	15264	WA	2020
OQ331221	15218	WA	2020
OR287917	15013	WA	2020
OR287918	15173	WA	2020
OR287919	15134	WA	2020
OR287927	15198	WA	2020
OR287948	15014	WA	2020
OR287976	15048	WA	2020
OR287984	15168	WA	2020
OR287985	15156	WA	2020
OP965711	15167	WA	2021
OR287986	15046	WA	2021
OR287987	15134	WA	2021
OR287988	15199	WA	2021
OP890317	15261	WA	2022
OP890331	15261	WA	2022
OP890332	15218	WA	2022
OQ024110	15238	MA	2022
OQ024120	15255	MA	2022
OQ171912	15239	MA	2022
OQ171931	15244	MA	2022
OR143176	15222	AZ	2022
OR143187	15224	AZ	2022
OR143219	15224	AZ	2022
OR143160	15220	AZ	2023
OR143161	15162	AZ	2023
OR143163	15221	AZ	2023
OR143171	15224	AZ	2023
OR143184	15226	AZ	2023
OR143185	15231	AZ	2023
OR522508	15172	OR	2023
OR522529	15197	OR	2023

bioRxiv preprint doi: https://doi.org/10.1101/2024.04.22.590570; this version posted April 23, 2024. The copyright holder for this preprint (which was not certified by peer review) is the author/funder, who has granted bioRxiv a license to display the preprint in perpetuity. It is made available under aCC-BY 4.0 International license.

OR601479	15197	WA	2023
OR601480	15197	OR	2023

Table S3: Annotated variations observed in the CDS of replication-associated genes (N, P, M2, L) of selected 109 RSV A sequences. An asterisk "*" indicates that the sequence has no variation in the gene when compared to the consensus sequence. **See Excel file uploaded**.

Table S4: Complete version of Table 4 showing variations observed in more than 2 sequences. Variations are arranged by their positions in the CDS and assigned to one of the groups R1-R6. **See Excel file uploaded**.

Table S5: Distribution of 109 sequences within each predicted groups including the year of sample collection and their locations in the US by states. UNKNs indicates that sequences are of unknown origin within the US.

**Group Name**	**Number of Sequences**	**Range of Years**	**US Locations**
**R1**	43	2012-2020	PA, TN, WA, UNKNs
**R2**	16	2021-2023	AZ, WA, UNKNs
**R3**	32	2015-2023	AR, AZ, MA, OR, TN, WA, UNKNs
**R4**	12	2016-2021	PA, UNKNs
**R5**	3	2019	UNKNs
**R6**	3	2012, 2014	OR, UNKNs

bioRxiv preprint doi: https://doi.org/10.1101/2024.04.22.590570; this version posted April 23, 2024. The copyright holder for this preprint (which was not certified by peer review) is the author/funder, who has granted bioRxiv a license to display the preprint in perpetuity. It is made available under aCC-BY 4.0 International license.

Table S6: Table showing assigned Nextstrain clade and Goya clade of each sequence compared to our predicted R1-R6 groups. Clades were determined using full-length sequences in the Nextclade tool. **See Excel file uploaded**.
